# Constraint based modeling of metabolism allows finding metabolic cancer hallmarks and identifying personalized therapeutic windows

**DOI:** 10.18632/oncotarget.24805

**Published:** 2018-04-13

**Authors:** Sergio Bordel

**Affiliations:** ^1^ Institute of Cardiology, Lithuanian University of Health Sciences, LT- 50162, Kaunas, Lithuania

**Keywords:** metabolism, metabolic models, therapeutic windows, RNA-seq, flux balance analysis

## Abstract

In order to choose optimal personalized anticancer treatments, transcriptomic data should be analyzed within the frame of biological networks. The best known human biological network (in terms of the interactions between its different components) is metabolism. Cancer cells have been known to have specific metabolic features for a long time and currently there is a growing interest in characterizing new cancer specific metabolic hallmarks. In this article it is presented a method to find personalized therapeutic windows using RNA-seq data and Genome Scale Metabolic Models. This method is implemented in the python library, pyTARG. Our predictions showed that the most anticancer selective (affecting 27 out of 34 considered cancer cell lines and only 1 out of 6 healthy mesenchymal stem cell lines) single metabolic reactions are those involved in cholesterol biosynthesis. Excluding cholesterol biosynthesis, all the considered cell lines can be selectively affected by targeting different combinations (from 1 to 5 reactions) of only 18 metabolic reactions, which suggests that a small subset of drugs or siRNAs combined in patient specific manners could be at the core of metabolism based personalized treatments.

## INTRODUCTION

It is well known that the metabolism of cancer cells differs from the metabolism of normal cells. The Warburg effect (aerobic lactic fermentation) has been known to be a characteristic of cancer cells for almost a century [1]. Metabolic reprogramming in cancer cells goes well beyond the Warburg effect and an increasing number of cancer specific metabolic hallmarks are being described [2–4]. As a result of that, there is also a growing interest in targeting metabolic enzymes for cancer therapy. A review by Vander Heiden [5] highlighted that there are currently around 30 metabolic enzymes considered to be targets of anti-cancer agents in different stages of development. These targets are related to metabolic processes such as nucleic acid synthesis, amino acid metabolism, lipid synthesis, glycolysis, TCA cycle etc. Indeed anti-folates have been used against many tumor types for almost half a century [6]. The previously mentioned review points out the main challenges in the development of metabolism based anticancer agents: “unwanted toxicity caused by the effects of agents targeting metabolic pathways in normal proliferating cells is likely to be a major challenge in the development of drugs that target proliferative cell metabolism. Several pathways often exist to generate the same metabolic end product, and redundant pathways that are present in normal cells but absent in cancer cells may provide a therapeutic window. However this same redundancy may also impair the efficacy of drugs in tumours that can use more than one pathway”. Fortunately, metabolism is the best known biological network and the problem of finding metabolic therapeutic windows, can be approached using Genome Scale Metabolic Models (GSMMs).

GSMMs [7, 8] are comprehensive compilations of all the metabolic reactions occurring in a particular organism. Each reaction is catalyzed by one or several enzymes, each of them coded by a gene. Thus a direct gene-protein-reaction connection is established. The structure of the metabolic network can be reflected by its stoichiometric matrix, which contains the stoichiometric coefficients of each metabolite in each reaction. Assuming that the concentration of each metabolite is in steady state, it is possible to define a space of feasible flux distributions and evaluate the metabolic capabilities of the cell (for example its capability to synthesize biomass building blocks). GSMMs can be used to compute distributions of metabolic fluxes using linear programming, this computation requires setting upper and lower boundaries for each metabolic flux as well as the choice of an objective function to be maximized (this is commonly referred as Constraint Based Modeling or CBM). The objective function is normally chosen to be the production of biomass, which is represented in the models as a biomass stoichiometric equation that includes the proportions of macromolecules forming a biomass unit. Vander Heiden, in his review [5] pointed out that “determining flux through the cancer cell metabolic network is likely to provide a better insight into successful enzyme targets”. Current methods to measure metabolic flux distributions such as the usage of ^13^C labeled substrates, are limited to a relatively small number of fluxes in the central carbon metabolism [9]. Experimentally measured fluxes can be used to constrain GSMMs and obtain estimations (averages and standard deviations) of fluxes not measured directly [10, 11]. However, direct or indirect measurements of metabolic fluxes are more costly and time consuming and less comprehensive than other high-throughput experimental techniques such as RNA-seq. The recent development of new high-throughput single cell RNA-seq methods [12] is likely to allow obtaining gene expression profiles of the different cell sub-populations in tumours using fast experimental protocols. For all these reasons we aimed to develop a computational method that allows finding personalized therapeutic windows using GSMMs and RNA-seq data. An intermediate stage in this method is the computation of putative metabolic flux distributions based in RNA-seq data alone. This is done by setting, for all the reaction rates in the model, upper bounds that are proportional to the expression levels of their associated genes. A similar approach (the PRIME method) consists in setting maximal boundaries to a set of reaction rates, based on gene expression microarrays [13]. In our approach every gene-associated reaction in the model is constrained based on the expression of its associated genes given as RPMK (reads per million per kilobase). A python library, pyTARG, has been developed in order to automatically constrain human GSMMs using RNA-seq data, provide estimations of metabolic flux distributions as well as sets of putative targets expected to impair the production of biomass building blocks in a target cell type (a cancer cell) while having lower effects on a reference cell type (in our case healthy mesenchymal stem cells).

Using RNA-seq data from healthy differentiated tissues and cancer cell lines, we estimated metabolic flux distributions and compared them in order to identify cancer metabolic hallmarks. Remarkably, already well described metabolic hallmarks such as higher glycolysis, glutaminolysis etc. were well reproduced in the estimated flux distributions, which supports the validity of the method presented here.

## RESULTS AND DISCUSSION

### Predictions of metabolic fluxes using constraint based modeling

**Figure 1:**
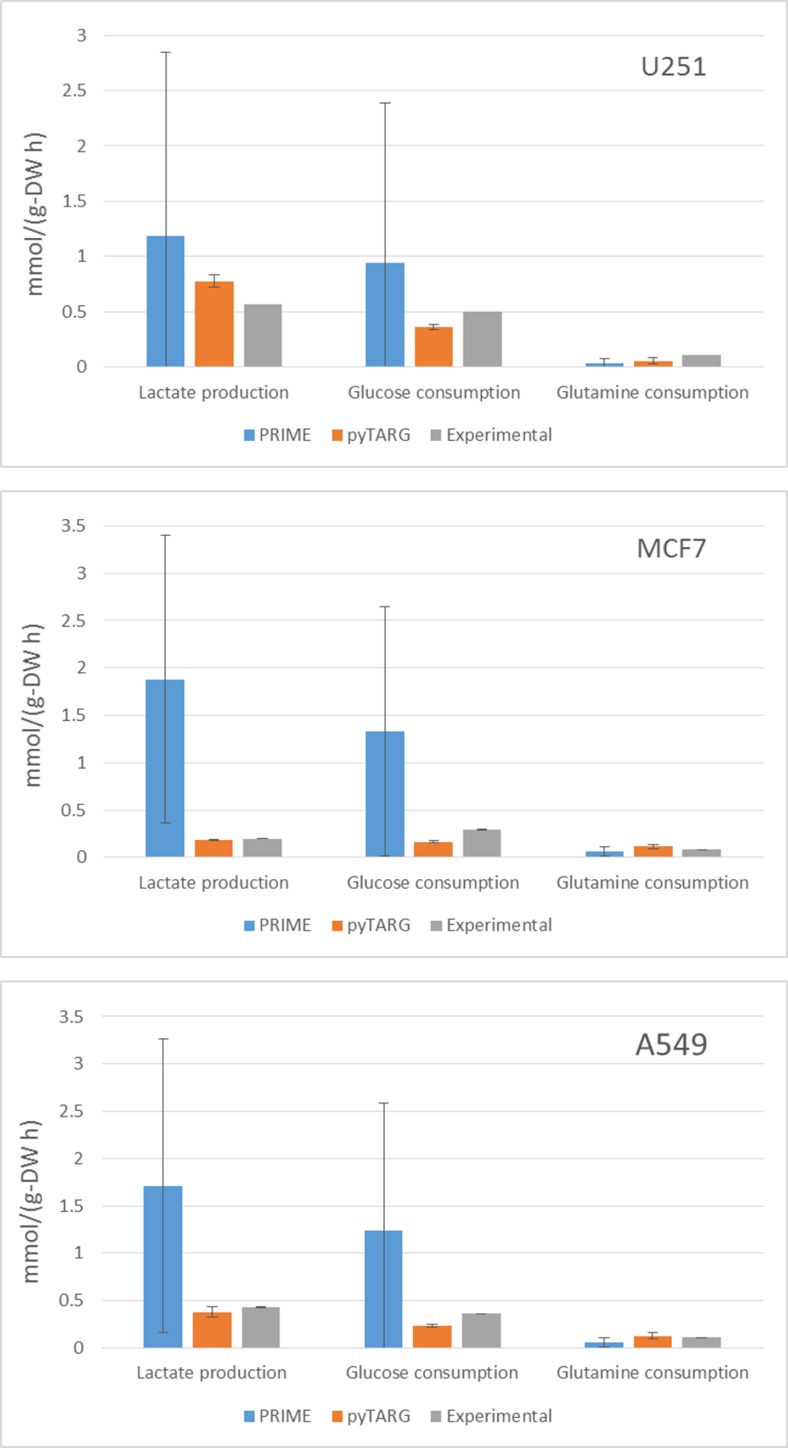
Average estimations of metabolic fluxes using pyTARG and PRIME and experimental metabolic fluxes, the error bars correspond to standard deviations

In order to assess if our constraint based modeling approach (pyTARG) can provide realistic estimations of metabolic fluxes and compare it to the existing method PRIME [13], we have selected 3 cell lines (MCF7, U251 and A549) for which RNA-seq data are available at the Human Proten Atlas (www.proteinatlas.org), and microarrays are available (as well as for all the NCI-60 cell lines) at CellMiner (https://discover.nci.nih.gov/cellminer/). The largest exchange metabolic fluxes of cancer cells are glucose and glutamine uptake and lactate secretion. We will benchmark our method based on its ability to quantify these fluxes. Experimental uptake rates for the selected cell lines are available in the literature [14]. The fluxes reported in the cited reference are given in moles per cell and GSMMs normally work with fluxes normalized by grams of biomass, therefore we need to obtain the typical cellular masses of each of the considered cell lines, which are also reported in the literature [15]. Both PRIME and pyTARG work by maximizing the biomass production rate after imposing constraints on the model (see methods). The flux distribution obtained after such maximization is just one among many possible flux distributions with the same optimal biomass production rate. In order to assess how accurately each of the methods is able to reproduce experimental flux distributions, a set of alternative optimal solutions was computed for each cell line as described previously [10], from these sets we computed averages and standard deviations for the predictors of lactate production, glucose consumption and glutamate consumption obtained using pyTARG and PRIME respectively. As it is shown in Figure 1 and Table 1, all the fluxes in all the cell lines are predicted more accurately by pyTARG, with especially large differences for lactate production and glucose consumption.

**Table 1:** Mean squared errors for each metabolic flux expressed in (mmol/g-DW h)^2^

U251	PRIME	pyTARG
**Lactate production**	3.14	0.045
**Glucose consumption**	2.30	0.0195
**Glutamine consumption**	0.0069	0.0030
**MCF7**		
**Lactate production**	5.14	0.0001
**Glucose consumption**	2.82	0.0164
**Glutamine consumption**	0.0031	0.0015
**A549**		
**Lactate production**	4.04	0.0052
**Glucose consumption**	2.60	0.0171
**Glutamine consumption**	0.0055	0.0018

The main difference between pyTARG and PRIME is that the first one constraints all the metabolic reactions based on the expression levels of their associated genes, while PRIME only constrains the reactions whose associated genes showed significant correlations with experimental growth rates. This leads to many reactions being left unconstrained (including reactions whose genes are completely non-expressed and which should be constrained to have a zero flux). Less constrains lead to more feasible solutions for the same biomass production rates and less accuracy in the prediction of metabolic fluxes.

### Identification of cancer metabolic hallmarks using constraint based modeling

**Figure 2:**
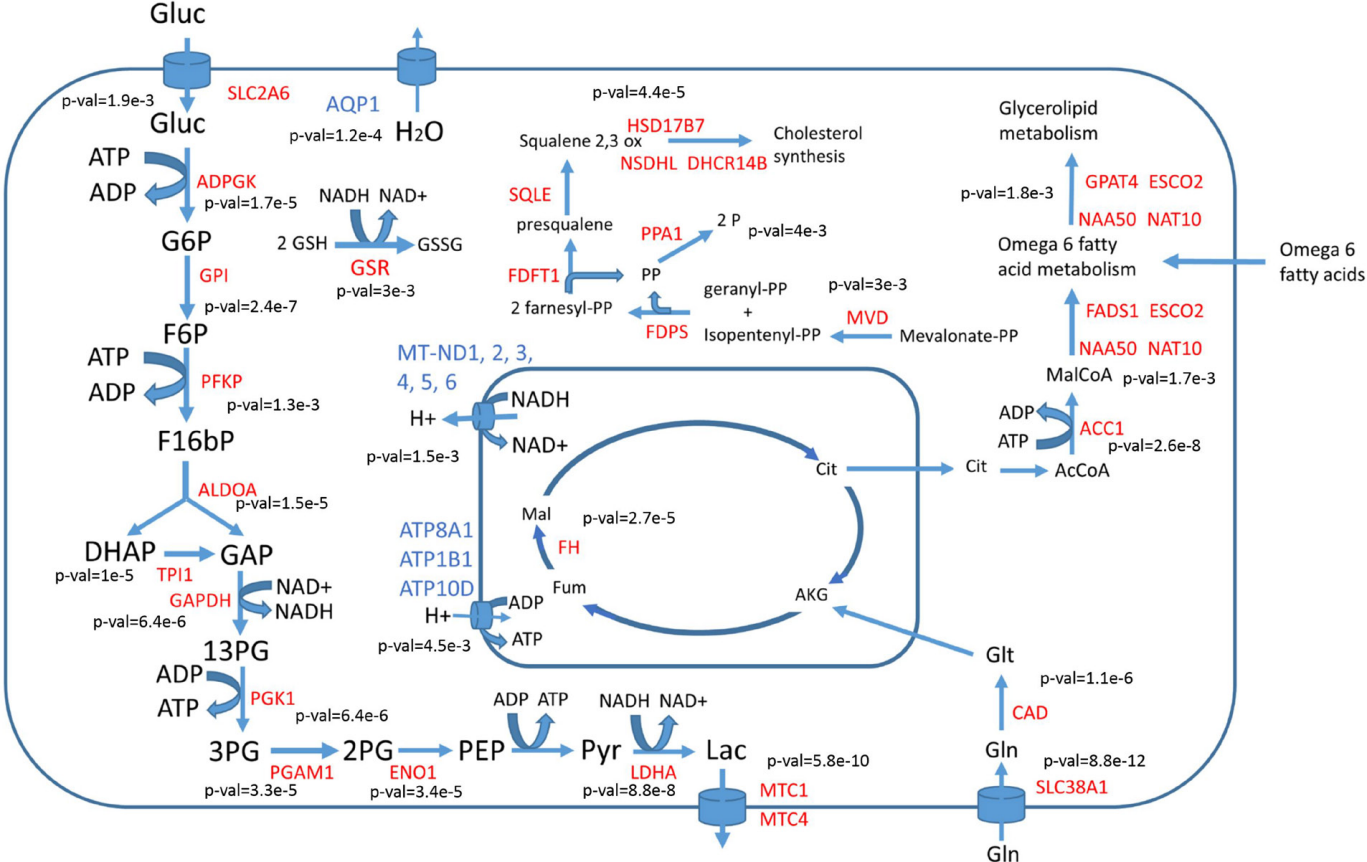
Main differentially used reactions between cancer cells and healthy tissues Enzymes with higher activity in cancer cells are labeled in red, those with lower activity in cancer cells are labeled in blue. *P*-values showing the significance of change are given near each reaction.

By using RNA-seq data, putative flux distributions for 34 different cancer cell lines and 26 healthy tissues were computed as described in materials and methods. RNA-seq data were obtained from the Human Protein Atlas (www.proteinatlas.org). A *t*-test was used to identify reactions showing statistically significant (with false discovery rates under 0.01 after correction for multiple testing) differences in metabolic fluxes (these reactions will be named “differentially used” reactions). In the same way differentially expressed genes (with 0.01 false discovery rates) were identified. Differentially used reactions associated to one or several differentially expressed genes were selected as cancer metabolic hallmarks. The list of identified metabolic hallmarks (with the corresponding *p*-values for each reaction and gene) is presented in the Supplementary File 1. Figure 2 summarizes the main hallmarks identified.

In full agreement with the Warburg effect, glucose uptake, glycolysis as well as lactic acid production and secretion were predicted to have higher metabolic fluxes in cancer cells compared to normal tissues. The respiratory chain was transcriptionally downregulated in cancer cells and also showed lower metabolic fluxes. Water transport by aquaporin 1 (AQP1) was downregulated in cancer cells, which is consistent with less water production due to lower activity of the respiratory chain. A concomitant increase in both gene expression and metabolic flux was observed in all the steps of glycolysis with the exception of pyruvate kinase. The expression of glycolytic enzymes is known to be induced by the PI3K/Akt signaling pathway, whose constitutive activation is one of the most common features in spontaneous human cancers [16]. Increased activity of the oncogene MYC and loss of the tumor suppressor TP50 have also been reported to result in increased glycolytic fluxes [17]. The hypoxia inducible factor HIF-1, which is downstream of the PI3K signaling pathway, also induces the expression of lactic acid transporters MTC1 and MTC4 [18]. Increased glutaminolysis, another key feature of cancer metabolism regulated by the oncogene MYC [4], is also predicted using our CBM approach. Glutathione reductase (GSR) was found to have higher metabolic activity in cancer cells, which is at the basis of the NADH depleting effect of vitamin C on cancer cells [17]. Metabolism of omega 6 fatty acids and arachidonic acid synthesis also appeared to be differentially upregulated in cancer cells compared to healthy tissues. This is consistent with the current knowledge of the impact of arachidonic acid metabolism on cancer cell proliferation [19]. Cholesterol biosynthesis was also predicted to be higher in cancer cells and its role as a suitable therapeutic window will be further discussed in the next section.

The expression levels and predicted metabolic fluxes for the glycolytic enzymes PGAM4 (phosphoglycerate mutase) and TPI1 (triosephosphate isomerase) are shown in Figure 3. More examples can be found in the Supplementary Figures 1–11.

**Figure 3:**
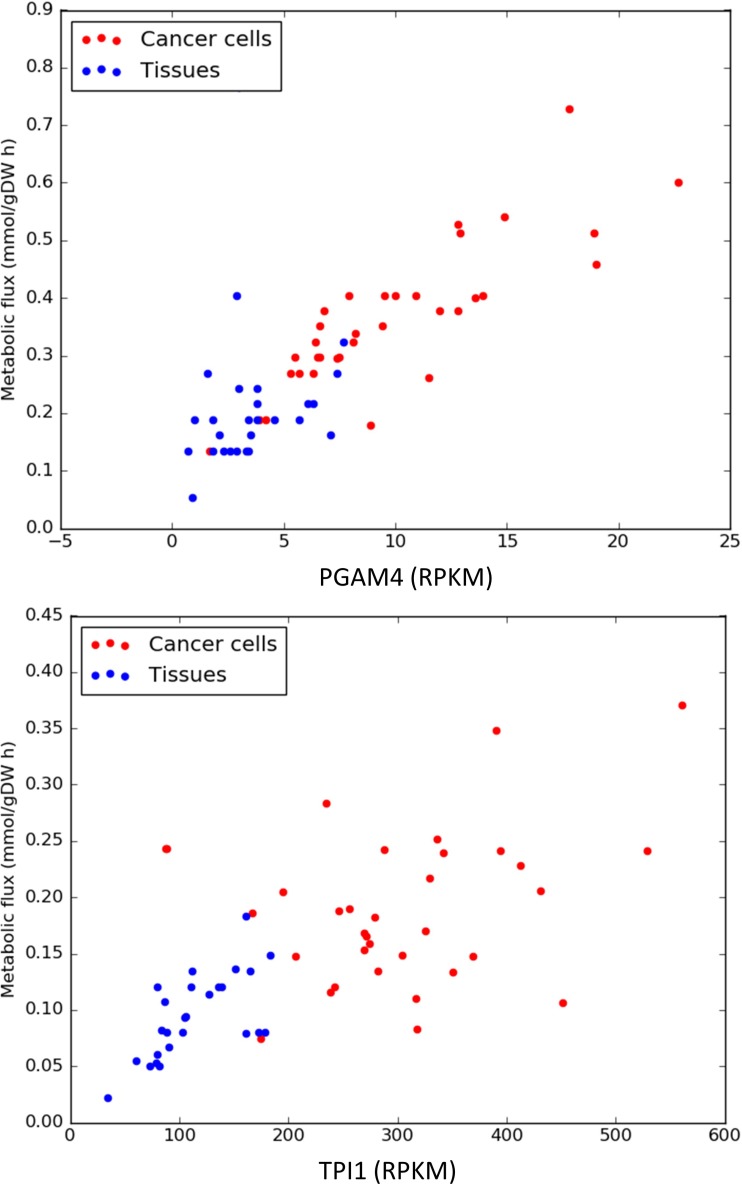
Expression levels and predicted metabolic fluxes (expressed in milimoles per hour and gram of cell biomass) for PGAM4 (phosphoglycerate mutase) and TPI1 (triosephosphate isomerase)

Overall we can conclude that constraining a human GSMM using RNA-seq data is a valid approach to compute metabolic flux distributions that mimic well known features of cancer metabolism. Therefore, CBM could be expected to be a suitable tool for predicting the effects of blocking certain metabolic reactions (pharmacologically or by gene silencing).

### Computational prediction of therapeutic windows

The main challenge to find new anticancer drug targets is the existence of deleterious effects on healthy cells, in particular on dividing healthy cells such as stem cells and progenitors. In order to test if the previously identified reactions can be targeted achieving negative effects on cancer cells while having lower effects on healthy cells, the metabolic flux through each target reaction was forced to have a maximal value equal to 0.1 times its original value (calculated using CBM). A new flux distribution was computed after imposing the mentioned constraint and the impact on growth on each cell line was reported as the ratio between the new estimated growth rate and the original one. A ratio of 0.1 means that biomass production is fully coupled to the targeted reaction. In the opposite extreme, a value of 1 would mean that there are alternative pathways able to fully compensate the drop in activity of the targeted reaction (see materials and methods for a full description). This was performed on the 34 cell lines previously used and 6 mesenchymal stem cell lines (3 from bone marrow and 3 form placenta) [20]. Mesenchymal stem cells were chosen because they share with metastatic cells the feature of having undergone Epithelial to Mesenchymal Transition, which makes them more similar to cancer initiating cells than other healthy dividing cells.

A *t*-test was carried out in order to identify reactions that have significantly higher effects on cancer cells compared to the 6 mesenchymal stem cells. The results (*p*-values, t-scores and average differences in effect) for each of the tested reactions are reported in the Supplementary File 2. The two most statistically significant reactions were found to be lactate dehydrogenase and lactic acid transport, which is consistent with the Warburg effect. Enzymes related to lactate metabolism (LDHA and MTC4) have been reported as suitable targets in multiple pre-clinical studies [21, 22]. Glucose uptake follows lactic acid production and secretion in statistical significance, which is consistent with the known increased dependence of cancer cells on glucose consumption. There are ongoing preclinical efforts to exploit glucose transporters as anticancer targets [23, 24]. Despite the clear differential effect on cancer cells compared to healthy stem cells, the simulations predict relatively low effects on the proliferation rates of cancer cells, with decreases of 20% for the most sensitive cell lines. This suggests that cell metabolism is robust enough to largely compensate the dependence of the cells on the mentioned reactions, therefore targeting lactate production or glucose uptake likely should be combined with other targets in order to achieve larger cytostatic effects. In contrast to other metabolic hallmarks, blocking cholesterol synthesis resulted in a drastic effect on the growth capabilities of most of the considered cancer cell lines (Figure 3), and left almost unaffected 5 of the 6 mesenchymal stem cell lines.

**Figure 4:**
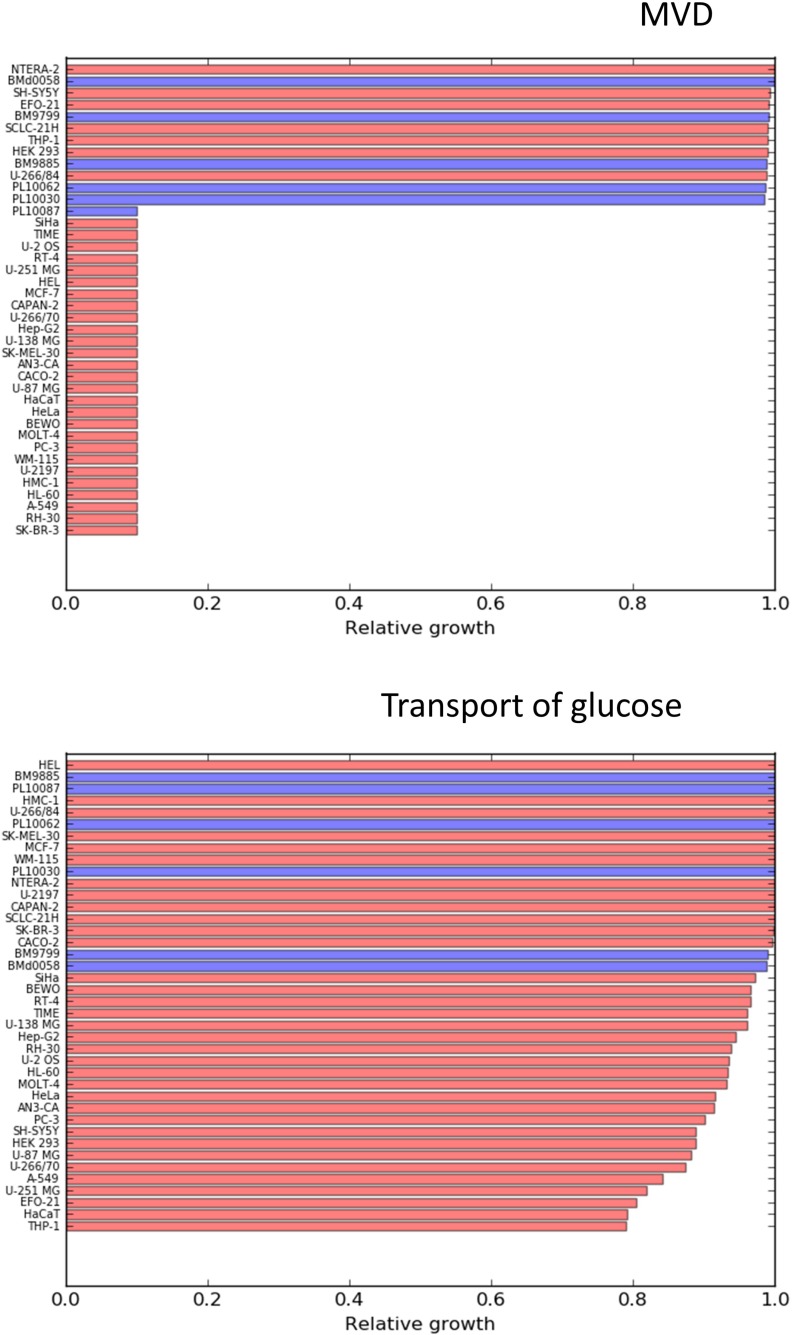
Relative effects on cancer cell lines (red bars) and healthy mesenchymal stem cells (blue bars) after constraining to 0.1 times their initial values, the flux through MVD (mevalonate diphosphate decarboxylase) and Glucose uptake

The effects of targeting MVD (mevalonate diphosphate decarboxylase) and glucose uptake are represented in Figure 4. The effects of more reactions are shown in the Supplementary Figures 12–15.

Deregulation of the mevalonate pathway and cholesterol metabolism in cancer, has already been described [25]. Overexpression of the enzyme HMG-CoA reductase has been reported to increase anchorage independent growth of malignant cells as well as the growth of xenograft tumors *in vivo* [26]. High amounts of stored cholesteryl esters in tumors are considered as hallmarks of cancer aggressiveness [27]. The potential therapeutic window offered by cholesterol biosynthesis is particularly interesting given the fact that there already exist approved inhibitors of cholesterol synthesis such as statins. The first anticancer clinical trials with statins were carried out already in 1996 and currently more than 18 clinical trials (phase I and II) have been carried out [28]. Preclinical experiments have shown anticancer activities (cytostatic and pro-apoptotic effects) of statins both in solid and liquid tumors. For example lovastatin (an inhibitor of the mevalonate pathway) has been shown to induce apoptosis in leukemia cells while keeping unaffected normal bone-marrow progenitors [29, 30]. This is a confirmation of the potential selective effects of targeting cholesterol biosynthesis. In a recent review [28] it is pointed out that it would be very beneficial to find markers allowing to identify which patients are sensitive to a treatment with statins. The cell lines in which biomass production was predicted to be fully coupled to cholesterol biosynthesis, were characterized by lacking the expression of the cholesterol transporter NPC1L1 and the lipoprotein lipase LPL, involved in the assimilation of lipoproteins from the blood stream. This makes these cells unable to incorporate external cholesterol and dependent on its biosynthesis. These two enzymes are therefore potential biomarkers for the sensitivity to a treatment with statins. Among the 34 considered cancer cell lines, 27 lacked expression of the mentioned enzymes and were predicted to be sensitive to impaired cholesterol metabolism. In order to check if it is possible to observe this phenomenon *in vivo*, expression profiles of NPC1L1 and LPL were downloaded from the TCGA Research Network (http://cancergenome.nih.gov/). Tumors are composed of many different cell types, including cells from tumor associated stroma, therefore using bulk gene expression data has large limitations, which are likely to be overcome with the development of high throughput single cell sequencing techniques [12]. However the data from the TGCA research network (see Supplementary Figures 27 and 28) revealed lower expression (more than a standard deviation lower than the corresponding healthy tissues) of NPC1L1 in: bladder urothelial carcinoma, cervical cancer, cholangiocarcinoma, head and neck squamous cell carcinoma, all the kidney cancer types, sarcoma and thymoma. LPL interestingly also appeared downregulated in bladder urothelial carcinoma, cervical cancer, all the kidney cancers and thymoma. Besides that, LPL was also downregulated in breast cancer, lung adenocarcinoma and lung squamous cell carcinoma. The mentioned tumors could be therefore more sensitive to treatments with drugs blocking cholesterol biosynthesis. Nevertheless, future treatments based on targeting metabolic pathways should be personalized based on RNA-seq data of individual patients.

### Computational choice of optimal target sets for individualized treatments

Efficient cancer treatments will require finding therapeutic windows that allow targeting selectively cancer cells while keeping the damage on healthy proliferating cells as low as possible. These therapeutic windows are likely different for each patient and should be determined by analyzing individual gene expression profiles (of tumor cells but also of healthy cells to be used as a reference). In this work, we have developed a heuristic algorithm implemented in the python programing language and contained in the library pyTARG (see materials and methods). This algorithm finds sets of metabolic reactions that, targeted simultaneously, lead to a reduction in the predicted biomass production rate down to a half of its initial value, while keeping as mild as possible the effects on a second cell type chosen as a reference.

In order to test pyTARG we found reaction sets targeting selectively each of the 34 cancer cell lines used in this article. The placental mesenchymal stem cell line PL10087 was used as a reference. This cell line was chosen because it is predicted to be sensitive to blocking cholesterol biosynthesis (differently from the other mesenchymal stem cells used in this study), therefore the algorithm will output targets outside the cholesterol synthesis pathways.

**Figure 5:**
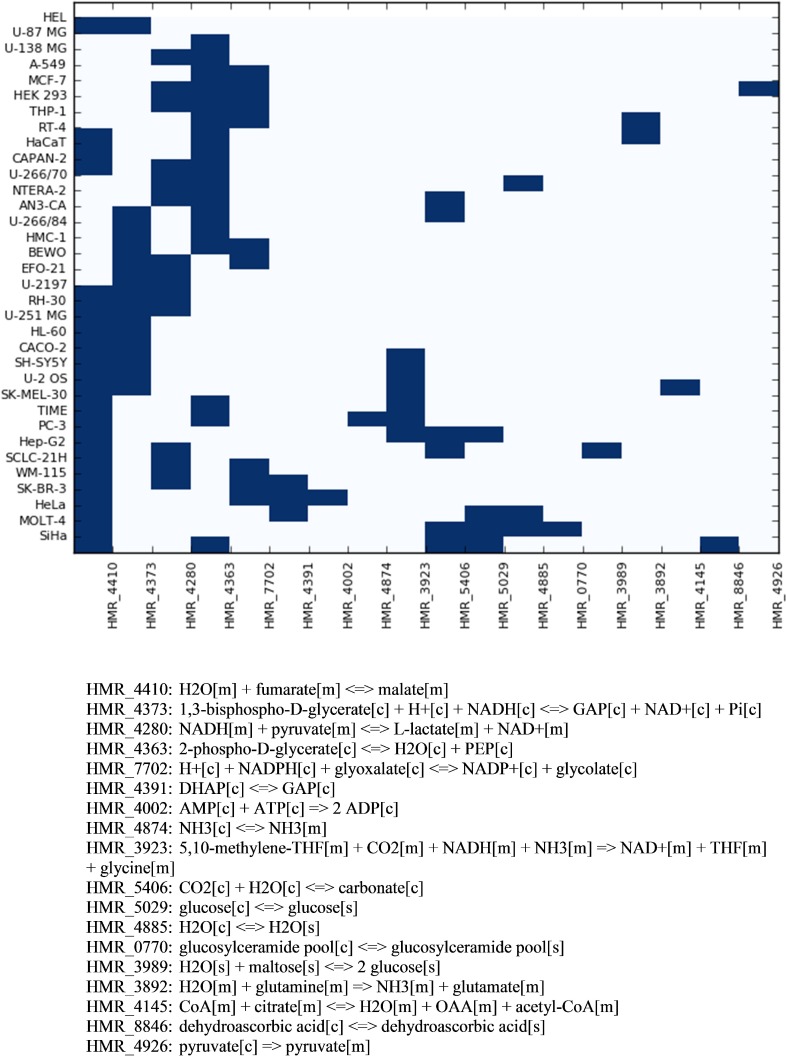
Combinations of reactions to be targeted in order to reduce selectively the proliferation rate of each cell line The horizontal axis of the heatmap shows the reaction identifiers in the Human Metabolic Reaction database. The stoichiometry of each reaction is shown below.

**Figure 6:**
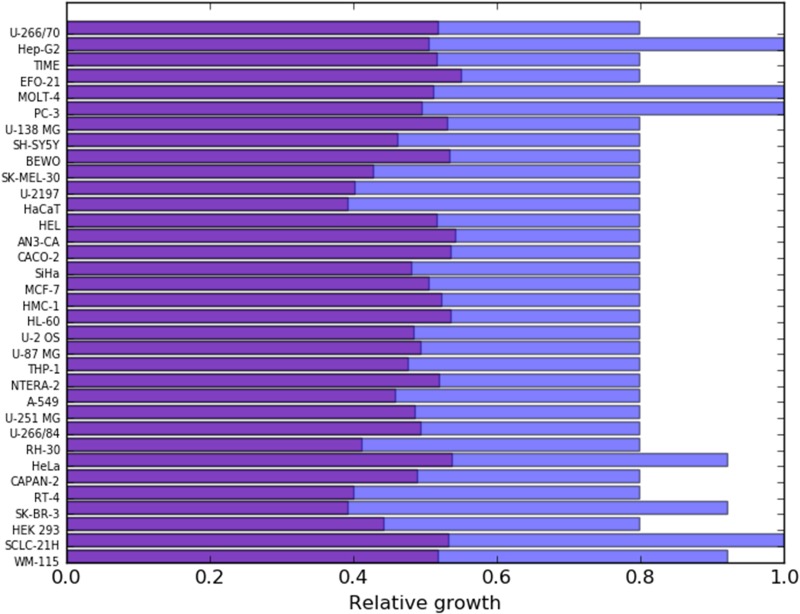
The purple bars show the relative growth of each cancer cell line after constraining the fluxes in their corresponding reaction sets (shown in Figure 5) to a maximum of 0.1 times their original fluxes The blue bars show the corresponding effects on the PL10087 mesenchymal cell line, which was used as a reference.

Figure 5 indicates which reactions sets were found by pyTARG for each of the tested cell lines. Figure 6 shows the relative effects on the targeted cell lines and the reference cell line.

Interestingly only 18 metabolic reactions appear in 34 different combinations despite the fact that the model contains several thousands of gene associated metabolic reactions. This means that the use of a rather limited set of enzyme specific inhibitors or small interfering RNAs, combined in a patient specific manner, could constitute the core of a metabolism-based therapeutic solution to cancer. All the reaction sets found by pyTARG include at least one of the glycolytic reactions identified in our previous analysis. This result is consistent with the Warburg effect and the current interest in developing glycolytic inhibitors as anticancer drugs [31]. Nevertheless, with few exceptions, glycolytic targets are not sufficient to cause drastic cytostatic effects and need to be combined with non-glycolytic reactions.

The reaction that appears most commonly among the sets of targets if fumarate hydratase (FH), one of the steps in the TCA cycle which was also revealed (in our previous analysis) to carry higher metabolic fluxes in cancer cell lines compared to healthy tissues. Interestingly, the FH gene has not been described as an anticancer target but as a tumor suppressor gene [32] whose loss of function due to germ line mutations results in hereditary leiomyomatosis and renal cell cancer (HLRCC) due to the accumulation of fumarate and succinate. Renal cancers with non-functional FH genes have been shown to be more dependent on glycolysis and particularly sensitive to its inhibition [33], which is fully consistent with our predictions.

### Evidence of compensatory responses in breast, prostate and lung cancers

The reactions in the sets found by pyTARG need to be targeted simultaneously in order to decrease the predicted proliferation rate down to 50% of its initial value. This means that the loss of activity of one single reaction in the set can be compensated by the increased activity of one or several of the remaining members of the set. Therefore it is reasonable to expect that tumors with a very low expression level of FH, will tend to show higher expression of one or several glycolytic enzymes.

Despite the already mentioned limitations of using bulk tumor RNA-seq data, we tested if the hypothesized compensatory effects are detectable in tumor samples. We downloaded RNA-seq data from the TCGA Research Network (http://cancergenome.nih.gov/), corresponding to the cohorts BRCA (breast cancer), PRAD (prostate cancer) and LUSC (lung squamous cell carcinoma). From each cohort, we selected the 20 samples with higher and lower expression levels of FH and performed a *t*-test for differential expression of glycolysis related genes. Significant compensatory effects (with *p*-values lower than 0.01) were observed for the glucose transporter gene SLC25A1 in the BRCA cohort and for the glucose transporter gene SLC2A9 in the PRAD cohort. No significant results were observed in the lung cancer cohort.

Following the same method, we tested for compensatory effects on the expression of aquaporins, which appeared in 3 of the reaction sets predicted by pyTARG and carbonic anhydrases, which appeared in 6 reaction sets. A significant overexpression of lactate dehydrogenase (LDHA) was observed in the three considered cancer types, compensating the loss of AQP5 (aquaporin 5). In the LUSC cohort, LDHA appeared to compensate also the downregulation of AQP2, 3 and 4. Our previous analysis (Supplementary Figure 10) showed that cancer cells show lower water transport rates than healthy tissues and a strong downregulation of AQP1, however, it appears that water transport mediated by other aquaporin genes is still important for cancer cells with moderate glycolytic rates. All these observations suggest that targeting alternative aquaporins such as AQP5, combined with LDHA, could constitute a selective therapeutic strategy against cancer. High expression of AQP5 has been linked to higher proliferation and metastasis in lung cancer [34]. In general, different aquaporins, have been associated to negative prognosis in different cancer types [35].

LDHA also appeared to compensate the downregulation of carbonic anhydrases: CA3 in breast and lung cancers, CA4 in prostate cancers and CA12 and 13 in lung cancers. The membrane bound CA9 and CA12 are the carbonic anhydrases that have received most attention as promising anticancer targets [36]. The purely stoichiometric nature of the model used in this article, which takes account only of the chemical reaction catalyzed by the enzymes, as well as the compensatory effects observed in the analyzed cancer cohorts, suggest that other carbonate anhydrases, cytoplasmic as well as membrane bound, could be suitable anticancer targets, in combination with LDHA or other enzymes involved in aerobic glycolysis.

The described compensatory effects are shown graphically in Figure 7 and in the Supplementary Figures 16–26.

**Figure 7:**
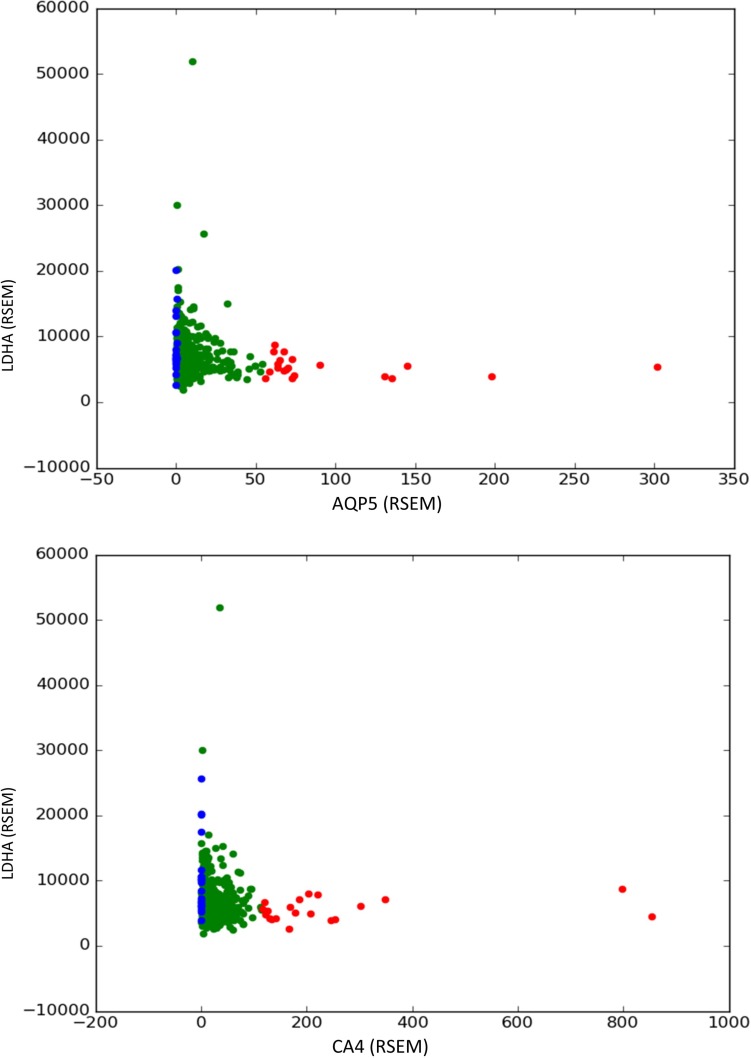
Compensatory effects of LDHA (lactate dehydrogenase) with AQP5 (aquaporin 5) and CA4 (carbonic anhydrase 4) found in prostate tumor samples A differential expression test for the expression of the gene in the y axis is performed between the 20 samples with higher expression of the gene in the axis x (red dots) and the 20 samples with lower expression of the gene in the axis x (blue dots). A significant overexpression in the second group indicates that downregulation of the gene in the axis x is compensated in some tumors by an upregulation of the gene in the axis y.

## CONCLUSIONS

It has been shown that human GSMMs constrained using RNA-seq data can be used to compute metabolic flux distributions that reflect well known metabolic hallmarks of cancer. The effects of blocking metabolic reactions in cancer cell lines and mesenchymal stem cells have been also tested computationally leading to the conclusion that cholesterol biosynthesis could be a highly selective therapeutic window to be targeted using statins or other drugs. An algorithm to identify personalized therapeutic windows have been developed, leading to the identification of groups of metabolic reactions with cytostatic effects (for 34 different cancer cell lines). These sets of reactions frequently involve glycolytic reactions combined with non-glycolytic ones such as fumarate hydratase, carbonic anhydrases or aquaporins. Compensatory effects were observed in tumor samples for some of the identified pairs of reactions. We can conclude that the use of stoichiometric metabolic models integrated with personalized gene expression data, could play a key role in the design of patient specific therapeutic strategies against cancer.

## MATERIALS AND METHODS

### Genome scale metabolic model

We have used a manually curated version of the Human Metabolic Reactions (HMR) database [37]. The model is presented in Excel format in the Supplementary File 3 and it has been deposited in SBML format in the database BioModels [38] and assigned the identifier MODEL1707250000. The model is allowed to uptake and secrete all the compounds present in HMR and whose uptake or secretion rates were measured experimentally in a previous study [14].

### RNA-seq data

Gene expression data for cancer cell lines (BioProject accession number PRJNA183192) as well as the healthy tissues included in the Human Proteome Atlas were downloaded from www.proteinatlas.org in the form of a comma separated file that contains the gene expression of each gene in each cell line (given as RMPK). This file was parsed using a customized python script (available upon request). RNA-seq fastq files for 6 different mesenchymal stem cell lines (3 from placenta and 3 from bone-marrow) were downloaded from https://usegalaxy.org/u/cic19/h/mesenchymal-stem-cells-rnaseq the reads were aligned on the complete list of human transcripts obtained from Ensembl BioMart using Bowtie2. The resulting alignment files were analyzed using a customized python script that is available upon request.

### Constraining GSMMs and computing flux distributions using PRIME

Upper bounds for the reactions whose associated genes are significantly correlated with growth rate were computed as described in the PRIME algorithm [13]. The reactions correlated with growth were found using microarray data and growth rates from CellMiner (https://discover.nci.nih.gov/cellminer/) corresponding to the NCI-60 cell lines. A false discovery rate of 0.05 was used. The selected reactions are reported in the Supplementary File 4 together with their correlation coefficients with the growth rate and their *p*-values.

### Constraining GSMMs and computing flux distributions using pyTARG

The model is constrained using the function fullconstrain from the pyTARG library. This library can be downloaded from https://github.com/SergioBordel/pyTARG. The function takes as input the model stored in BioModels after being imported using the python library COBRApy [39]. The RNA-seq data are provided to the fullconstrain function as a python dictionary whose keys are Ensembl gene identifiers and contain the expression of each gene reported in reads per million and kilo-base (RPMK). A short tutorial describing the use of all the functions in pyTARG is included in the Supplementary Material.

Each gene-associated reaction in the model is constrained to have a maximal value (or minimal if the reaction proceeds in the negative sense) proportional to the expression level of its associated gene. When a reaction is associated to several genes the highest expressed gene is chosen. Reactions not associated to any gene are left unconstrained. The proportionality constant between the gene expression and the upper boundary for the fluxes is a pure phenomenological constant chosen to reproduce the experimental growth rate of the cell line A549. The chosen proportionality constant was 0.0027 mmol g-DW^-1^h^-1^ times the expression level of the most abundant enzyme associated to each reaction (measured in RPKM). The boundaries were set in a discrete way (by rounding up the expression levels to their upper multiple of 10), this helped to avoid numerical problems while performing linear optimization.

After constraining the model and setting an objective function, the flux distribution can be computed using the function flux of the pyTARG library, which itself relies on the COBRApy library. The computed flux distribution corresponds to maximizing the objective function while keeping the total sum of metabolic fluxes as low as possible.

In this article we have always used the rate of biomass production as objective function. Biomass production is linked to proliferation rate, which is the main phenotype relevant to cancer that can be directly linked to a metabolic flux. On the other hand, the effects of blocking a metabolic reaction on other relevant phenotypes such as ATP production rate or NADH production rate (which could be linked to apoptosis for example) will be also observable on the biomass production rate. Interestingly, 26 of the healthy tissues form the Human Proteome Atlas were able to produce all the biomass building blocks necessary to proliferate. This does not mean necessarily that they proliferate, which would require that these building blocks are actually assembled into macromolecules and all the checkpoints of the cell cycle are being transited by the cells. Indeed we observed that most of the genes whose expression is correlated with growth rate are not metabolic genes but cell cycle components and ribosomal proteins [40]. Nevertheless we used the computed flux distributions as reasonable estimations for the metabolism of healthy tissues and this allowed us to retrieve all the well-known hallmarks of cancer metabolism previously discussed.

### Computing the effects of targeting metabolic reactions

The function *block* of pyTARG constrains the fluxes of one or several metabolic reactions to 0.1 times their original values and computes a new flux distribution after imposing the constraints. It outputs the ratio between the value of the objective function after and before imposing the new constraints. A ratio of 0.1 would mean that the objective function is fully coupled to at least one of the constrained reactions while higher values mean that the metabolism of the cell is theoretically able to compensate the new constrain by using alternative pathways. We chose to constrain the targeted reactions to 0.1 times their original value and not to zero, in order to account for the fact that in a real setup the reaction would not be fully blocked (pharmacologically or using siRNAs). Also in some cases, the metabolic flux of essential reactions (that result in a zero value of the objective function when fully removed from the model) can be strongly reduced without drastic effects on the objective function.

### Finding personalized therapeutic windows

The function personal of the pyTARG library implements a heuristic algorithm that aims to find a set of metabolic reactions with cytostatic effects on a target cell type while minimizing the effects on a healthy reference cell line. The inputs to the function are two models in COBRApy format, constrained using RNA-seq data for the target and the reference cell lines respectively. The algorithm starts by computing flux distributions for each of the two cell types. For each reaction in the model we take the difference between the flux in the target cell and the flux in the reference cell. The reactions are ranked in decreasing order starting from those with larger differences. Once the reactions have been ranked, the algorithm constrains the first reaction to 0.1 times its original flux in both cells. If the relative growth rate (in case the objective function is biomass production) on the target cell line is lower than 0.9 and the difference between relative growth rates between the target and the reference is higher than 0.05, the reaction is selected and the flux constraint is kept. If the conditions are not satisfied, the constraint is released and the same is repeated with the second reaction. The algorithm proceeds testing reactions until the relative growth rate of the target cell line drops to 0.5. The output of the function is a python list with the identifiers of the selected reactions. The chosen parameters of the algorithm have led to results involving relatively few reactions (from 1 to 5) as it is shown in Figure 5 and resulting in large differences in relative effects between the target and the reference cell lines (Figure 6).

## SUPPLEMENTARY MATERIALS FIGURES










