# Supplementary material for: Constraint based modeling of metabolism allows finding metabolic cancer hallmarks and identifying personalized therapeutic windows
Source: Oncotarget. 2018 Apr 13;9(28):19716–29. doi: 10.18632/oncotarget.24805 (PMC5929420; doi:10.18632/oncotarget.24805)
Supplement: Supplementary file 1 [file oncotarget-09-19716-s001.pdf]

Constraint based modeling of metabolism allows finding metabolic cancer hallmarks and identifying personalized therapeutic windows

SUPPLEMENTARY MATERIALS

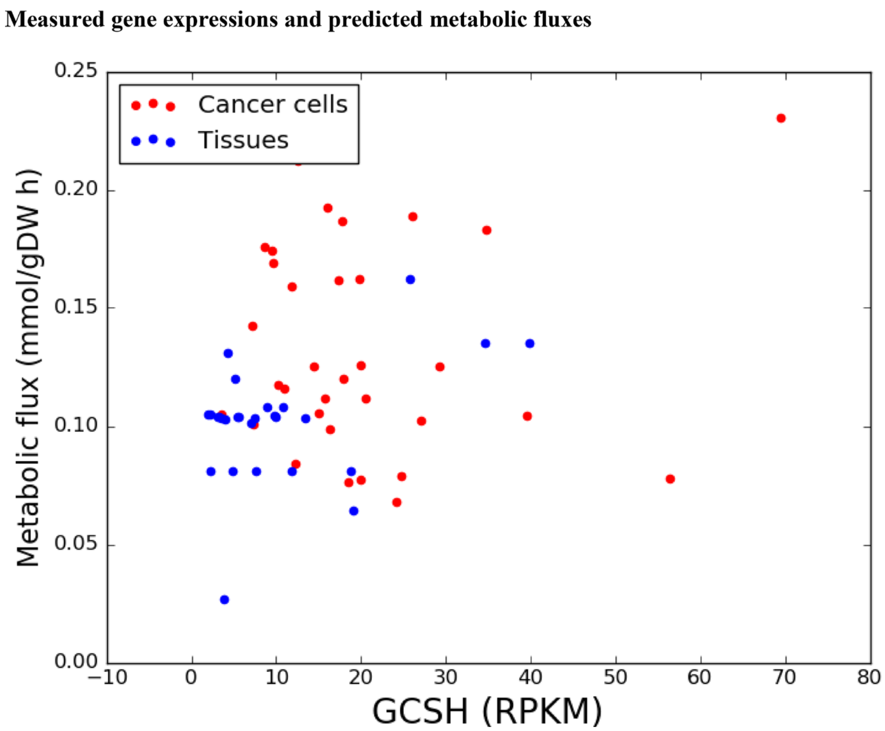

Supplementary Figure 1: Glycine cleavage system protein H.

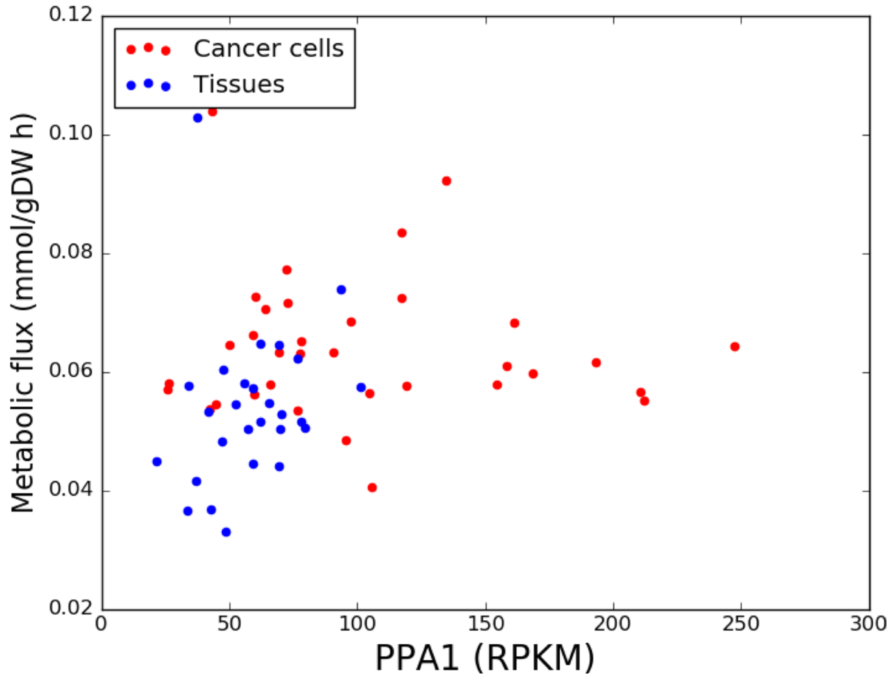

Supplementary Figure 2: Pyrophosphatase (Inorganic) 1.

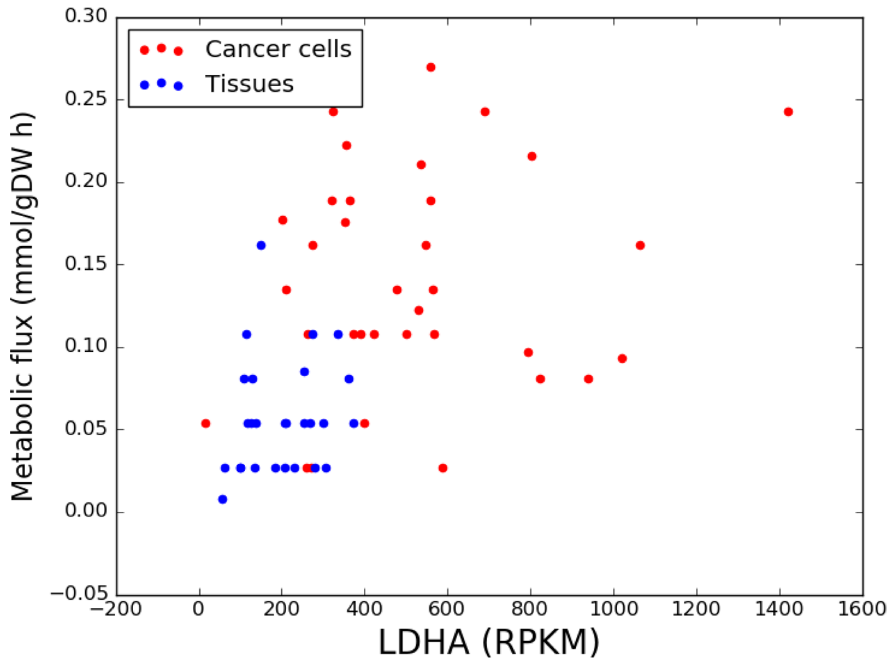

Supplementary Figure 3: Lactate dehydrogenase A.

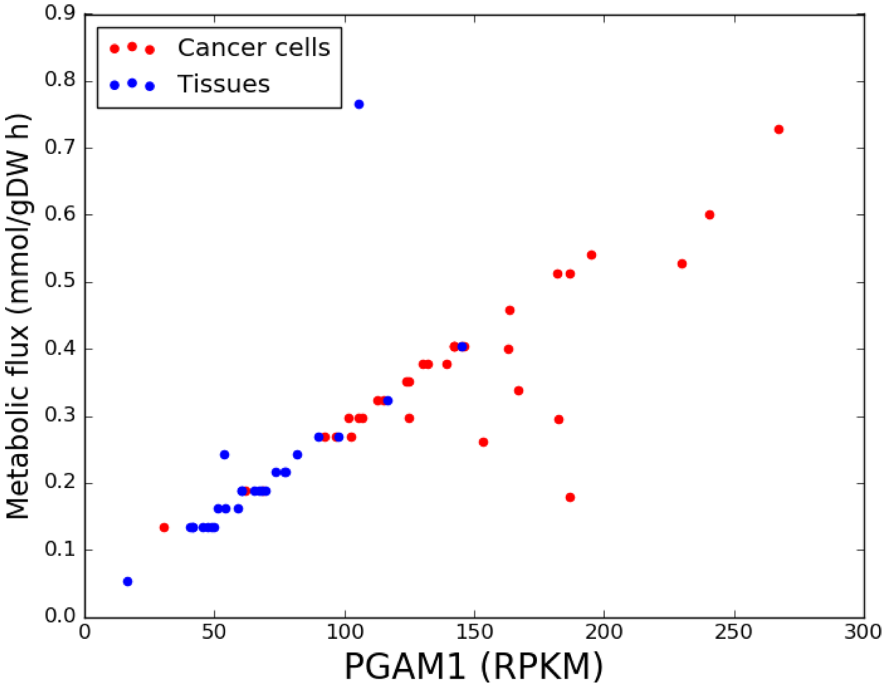

Supplementary Figure 4: Phosphoglycerate Mutase 1.

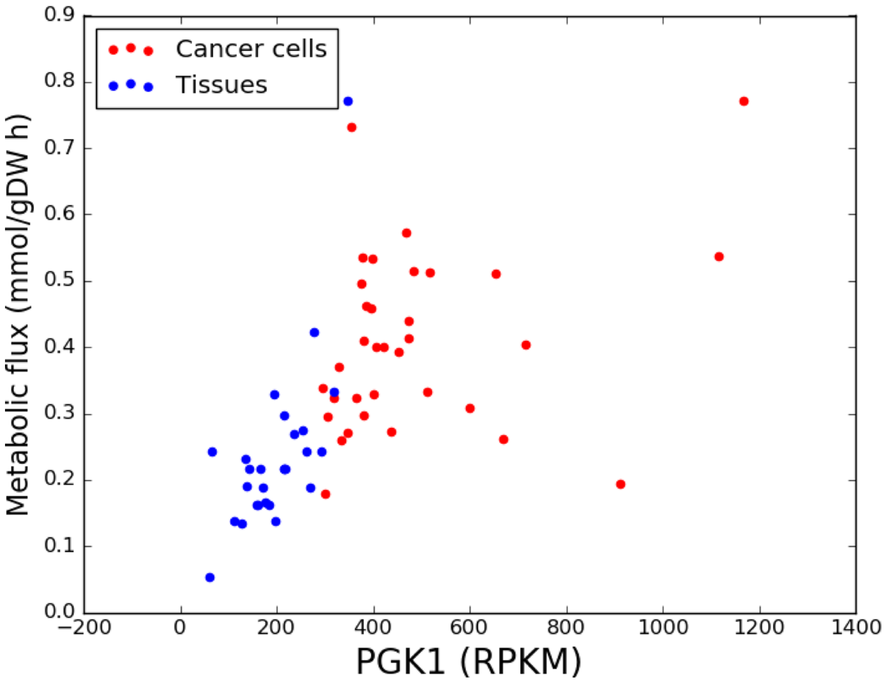

Supplementary Figure 5: Phosphoglycerate Kinase 1.

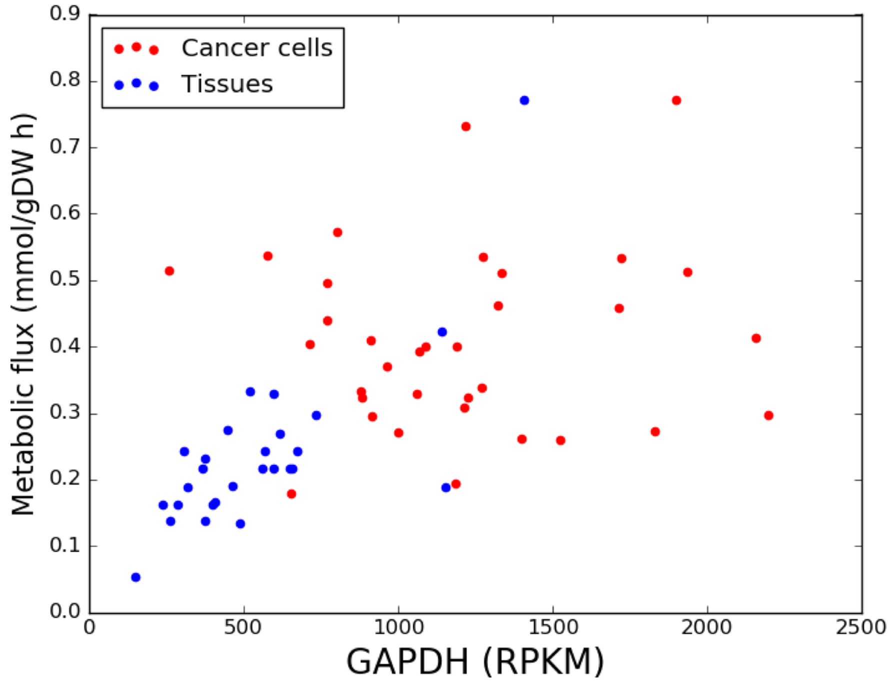

Supplementary Figure 6: Glyceraldehyde-3-Phosphate Dehydrogenase.

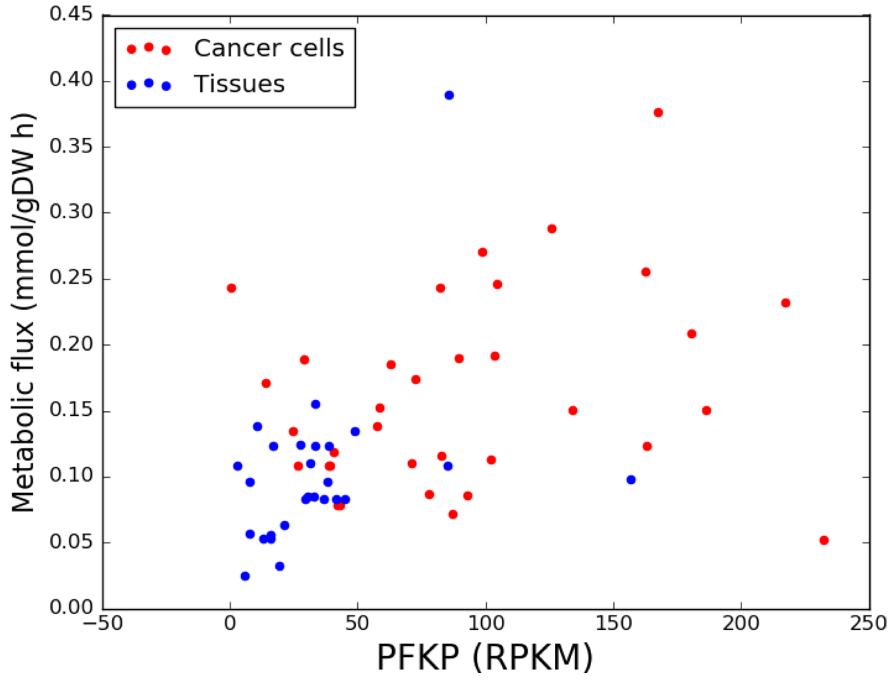

Supplementary Figure 7: Phosphofructokinase, Platelet.

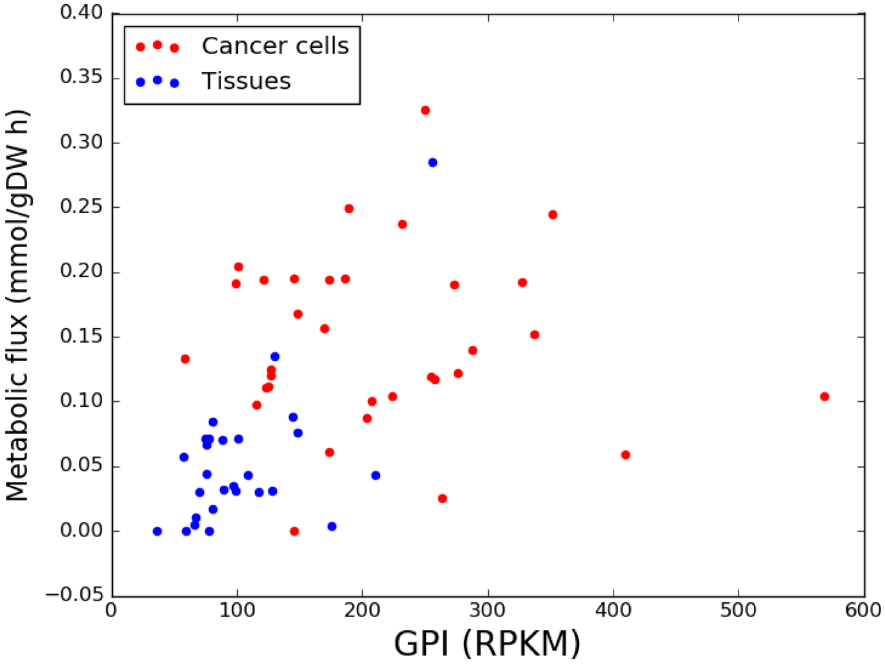

Supplementary Figure 8: Glucose-6-Phosphate Isomerase.

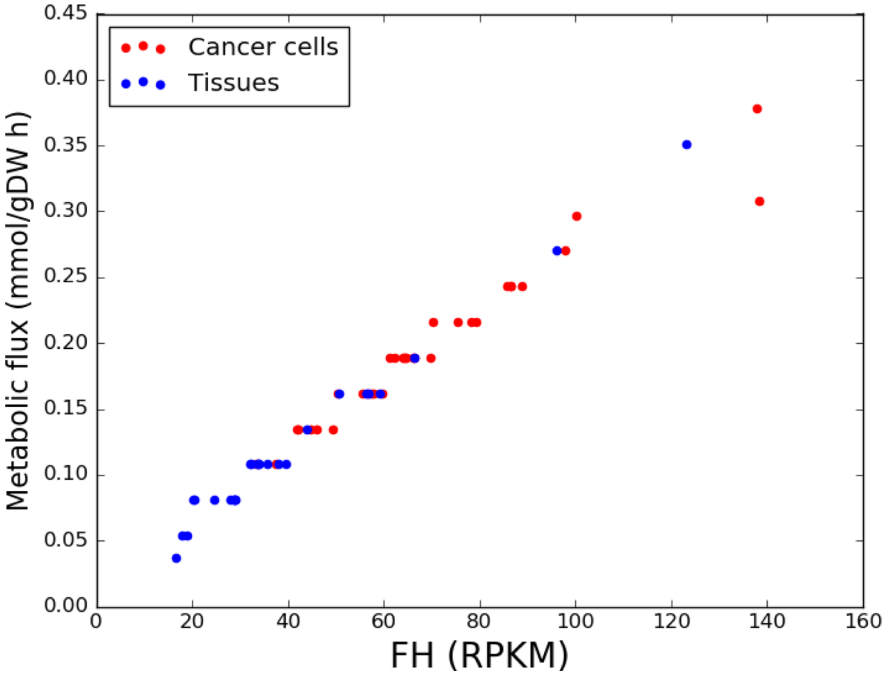

Supplementary Figure 9: Fumarate Hydratase.

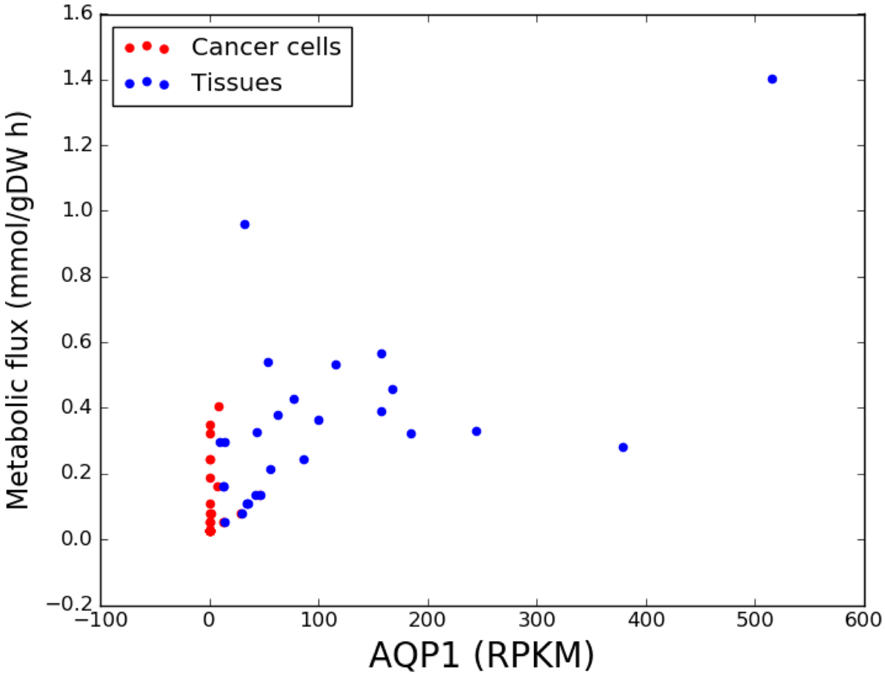

Supplementary Figure 10: Aquaporin 1.

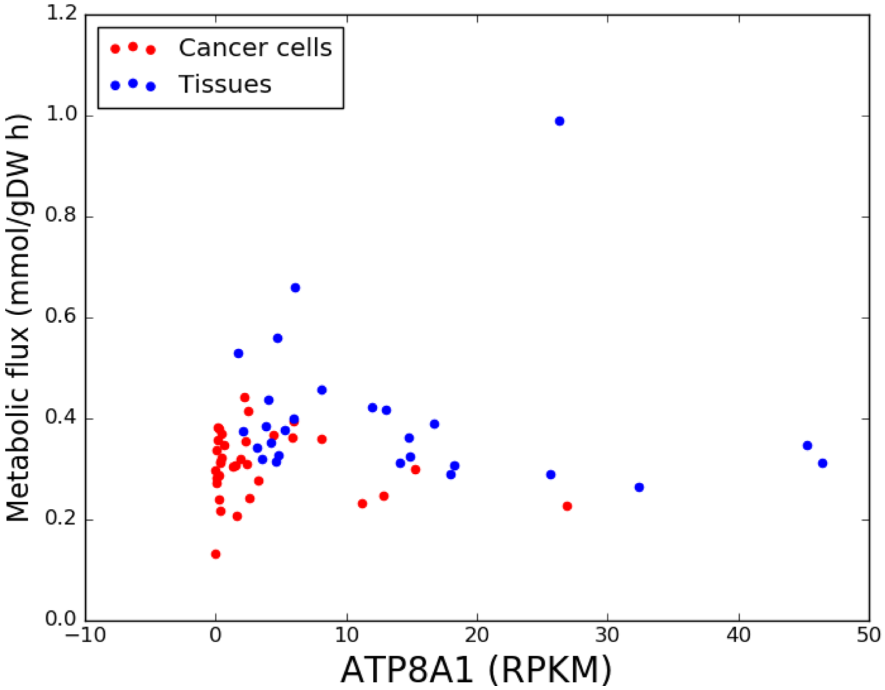

Supplementary Figure 11: ATPase Phospholipid Transporting 8A1.

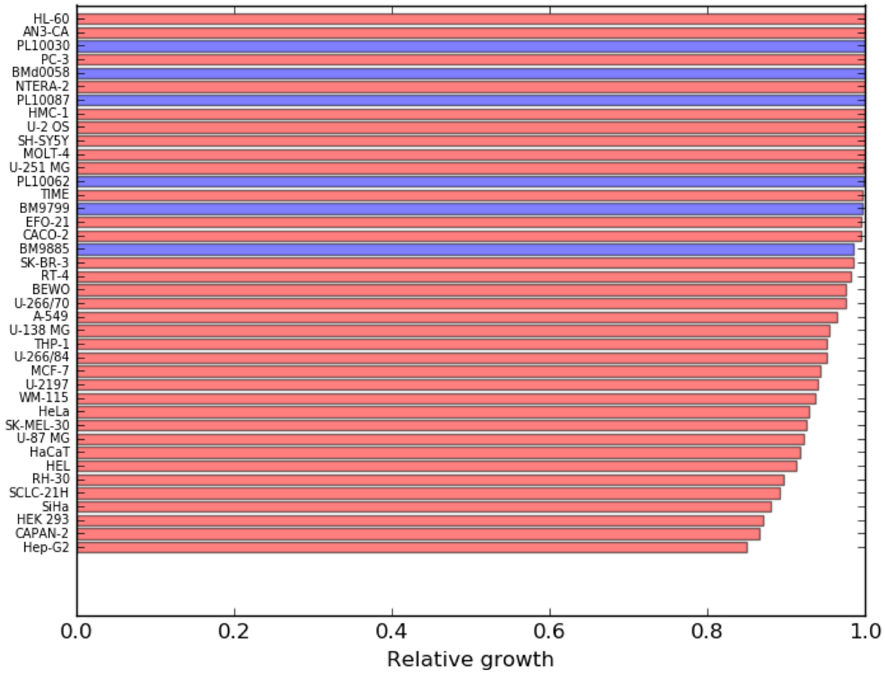

Supplementary Figure 12: Lactate production.

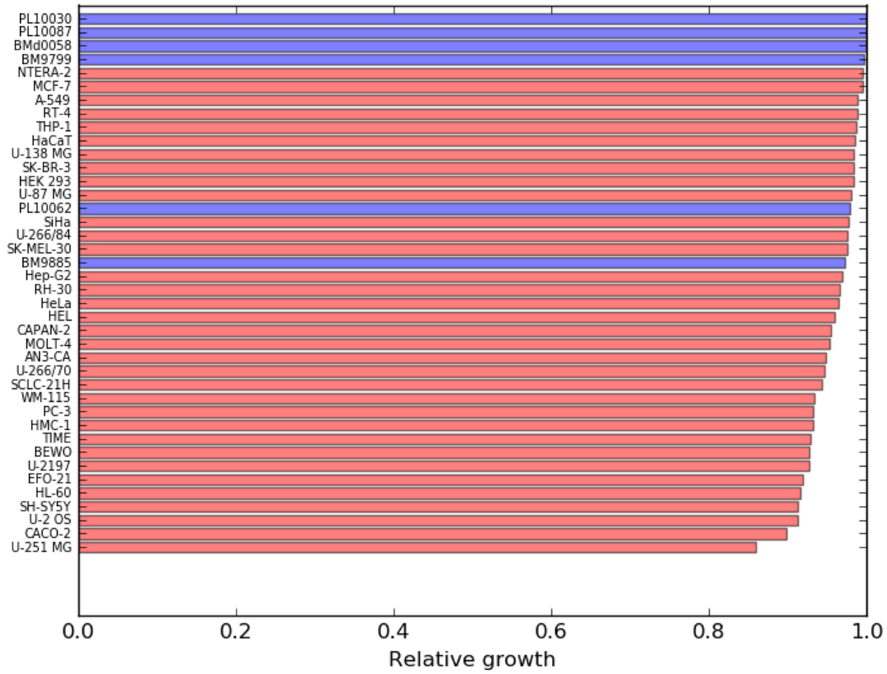

Supplementary Figure 13: Glycine cleavage system.

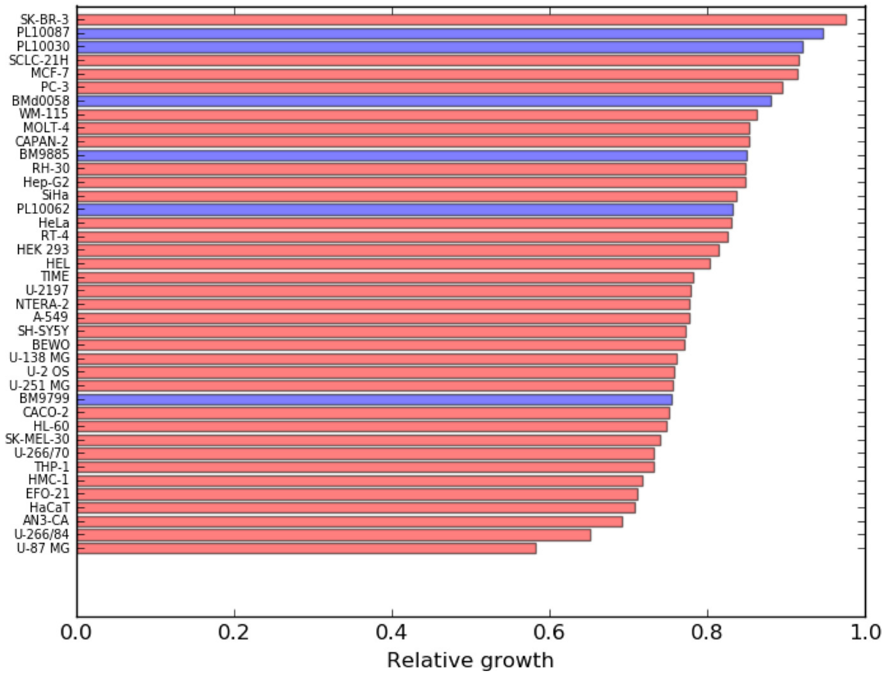

Supplementary Figure 14: Triosephosphate isomerase.

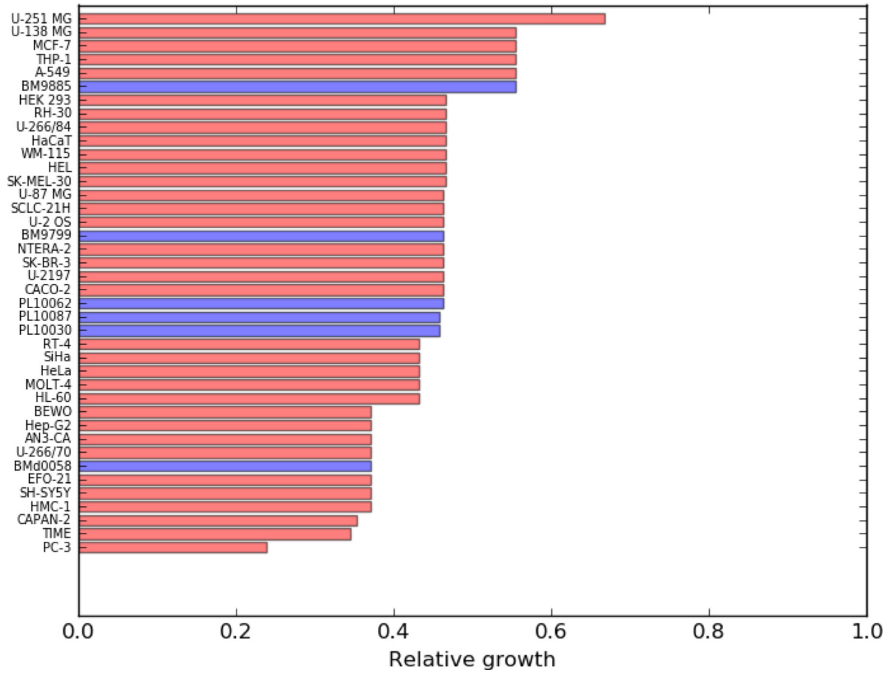

Supplementary Figure 15: Glycerol-3-Phosphate Acyltransferase.

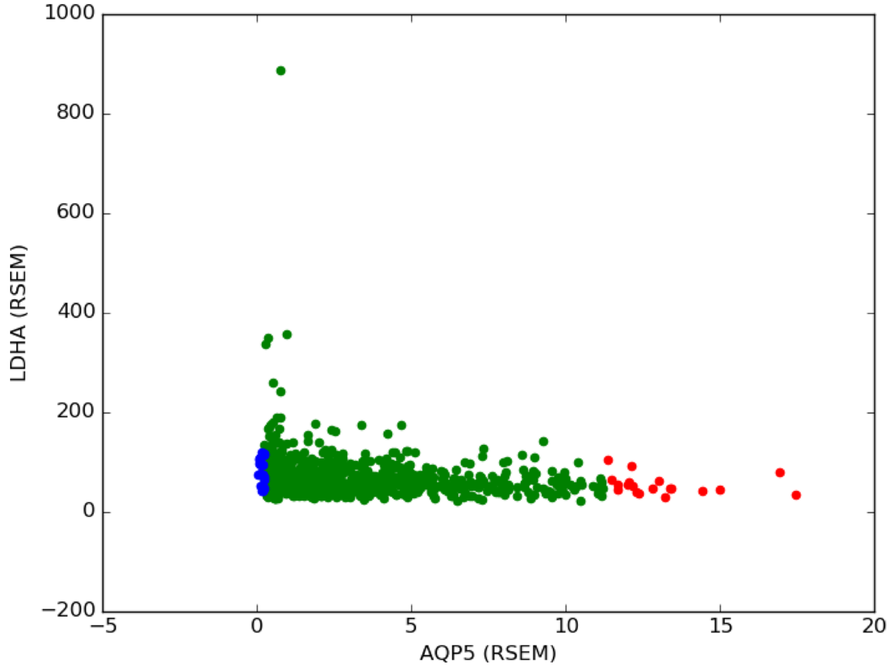

Supplementary Figure 16: Expression of the LDHA gene versus the expression of AQP5 in breast tumor samples.

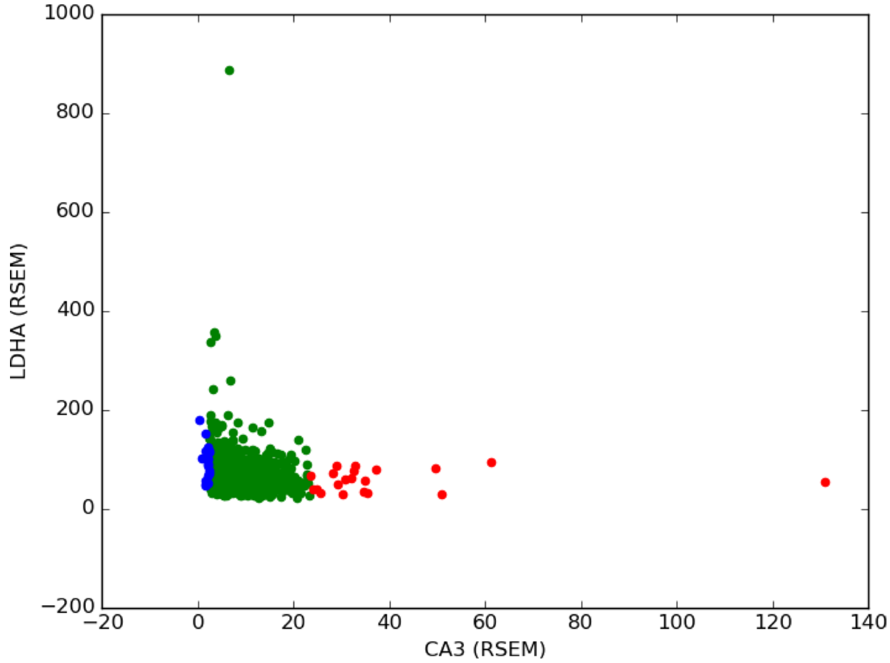

Supplementary Figure 17: Expression of the LDHA gene versus the expression of CA3 in breast tumor samples.

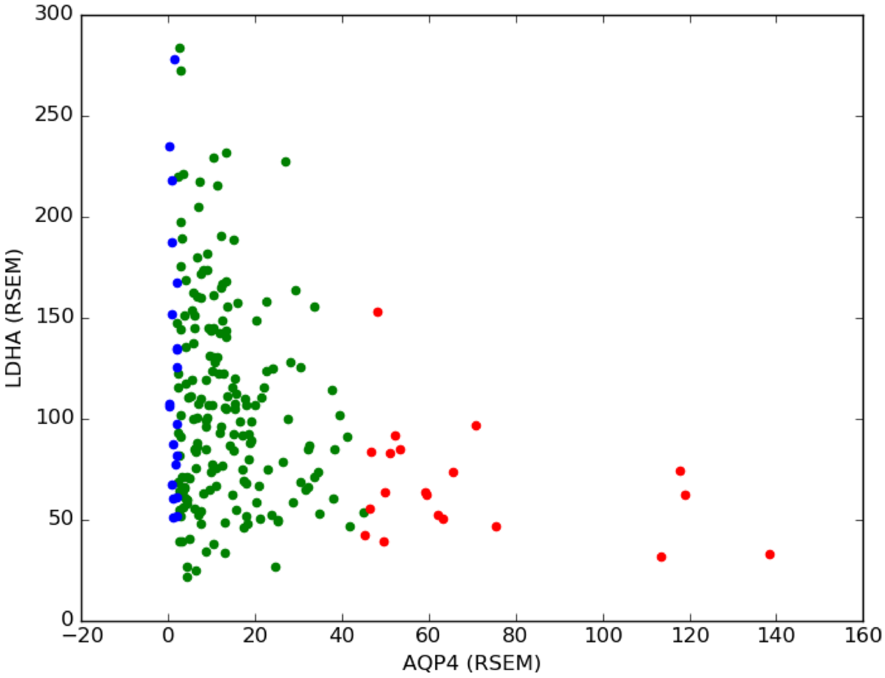

Supplementary Figure 18: Expression of the LDHA gene versus the expression of AQP4 in lung tumor samples.

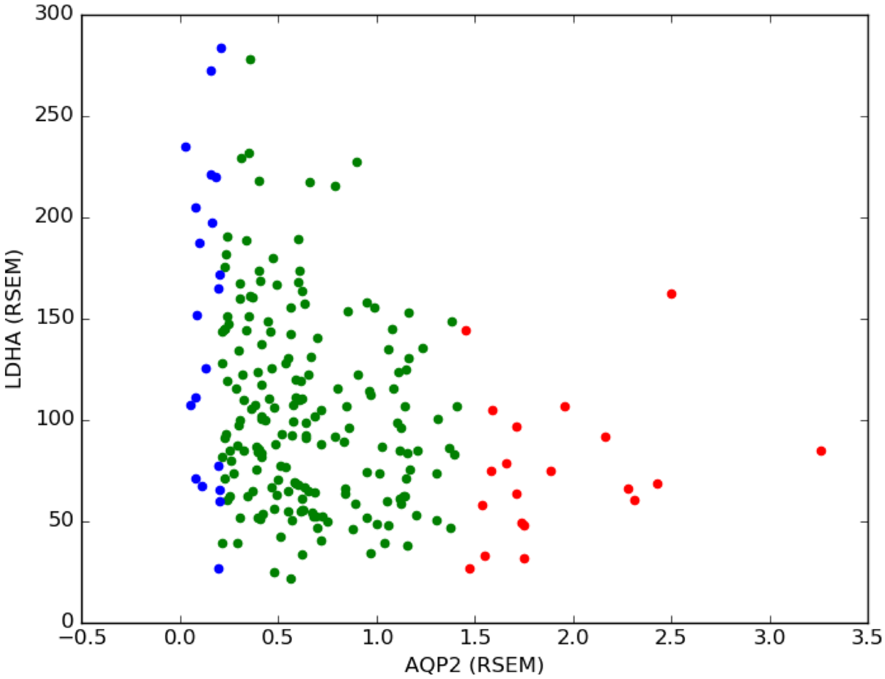

Supplementary Figure 19: Expression of the LDHA gene versus the expression of AQP2 in lung tumor samples.

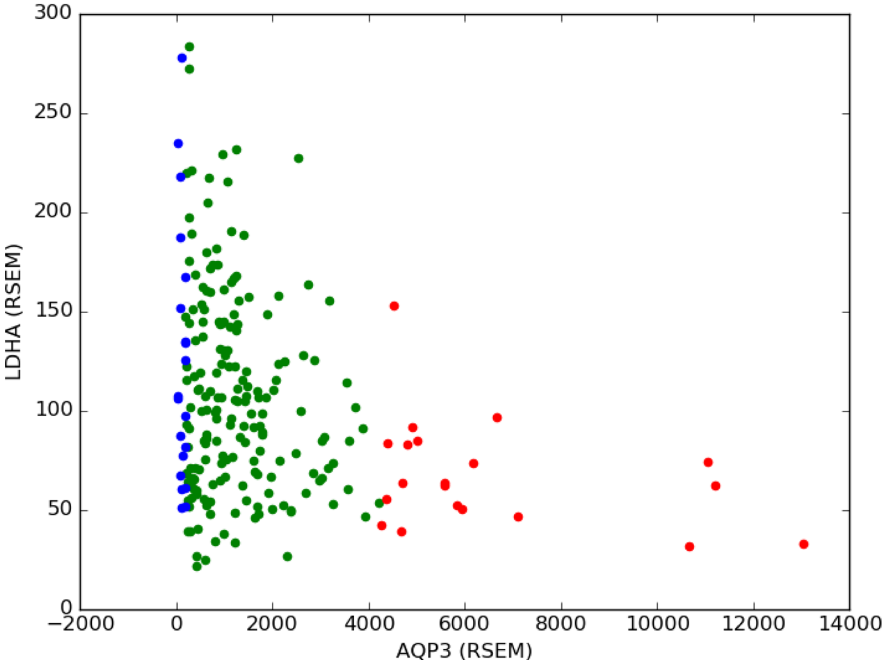

Supplementary Figure 20: Expression of the LDHA gene versus the expression of AQP3 in lung tumor samples.

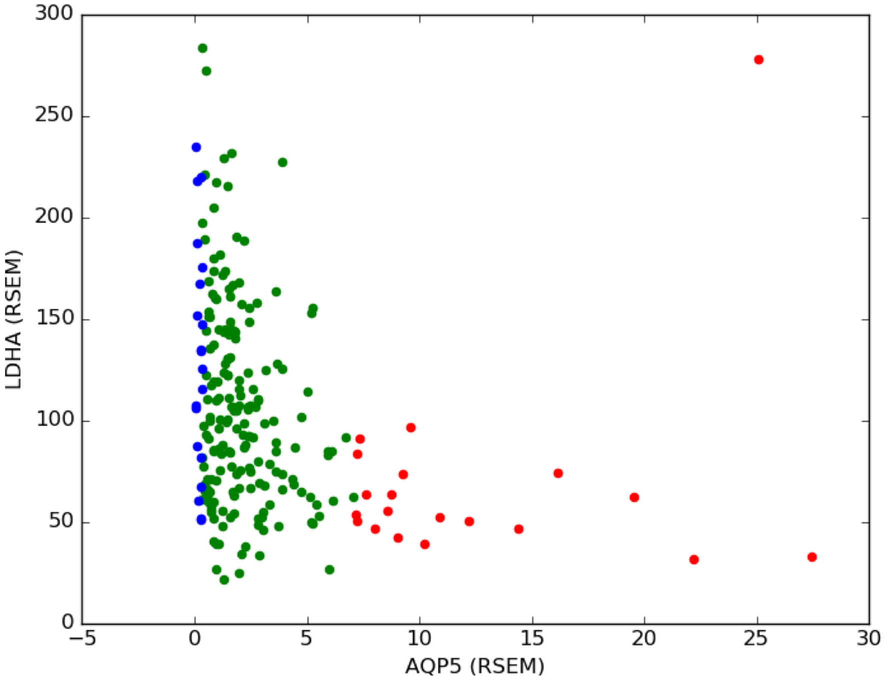

Supplementary Figure 21: Expression of the LDHA gene versus the expression of AQP5 in lung tumor samples.

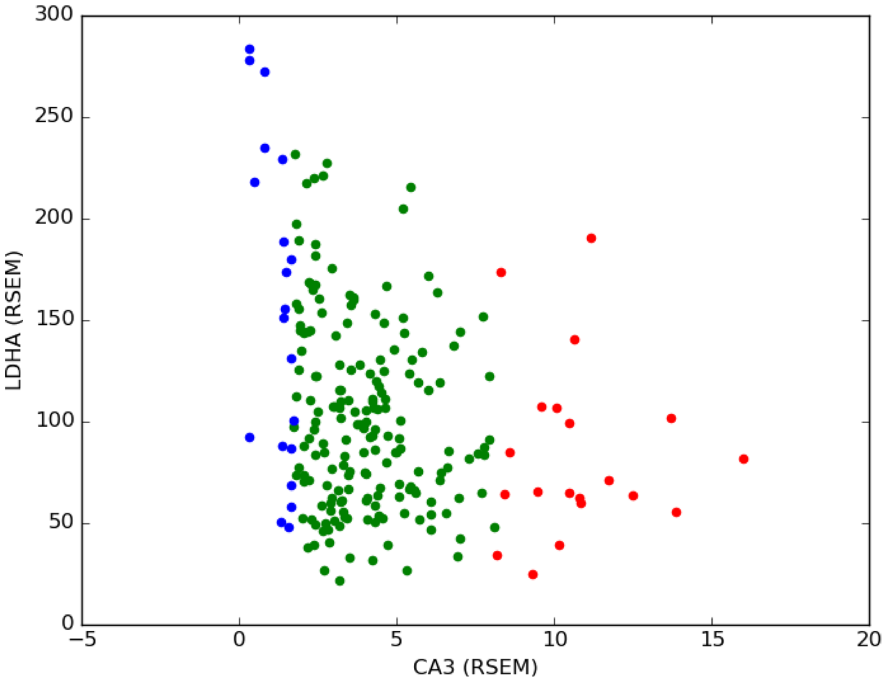

Supplementary Figure 22: Expression of the LDHA gene versus the expression of CA3 in breast tumor samples.

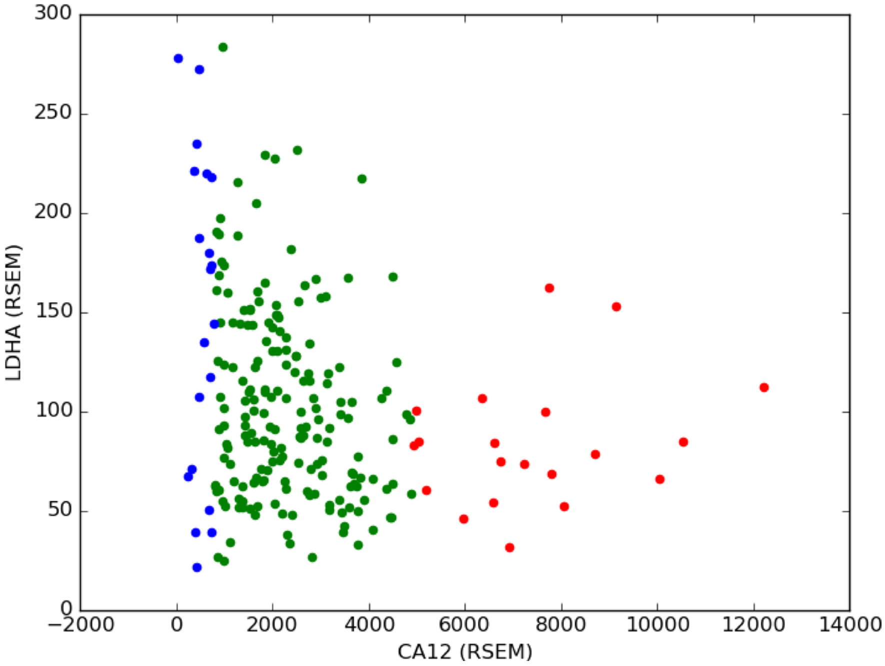

Supplementary Figure 23: Expression of the LDHA gene versus the expression of CA12 in lung tumor samples.

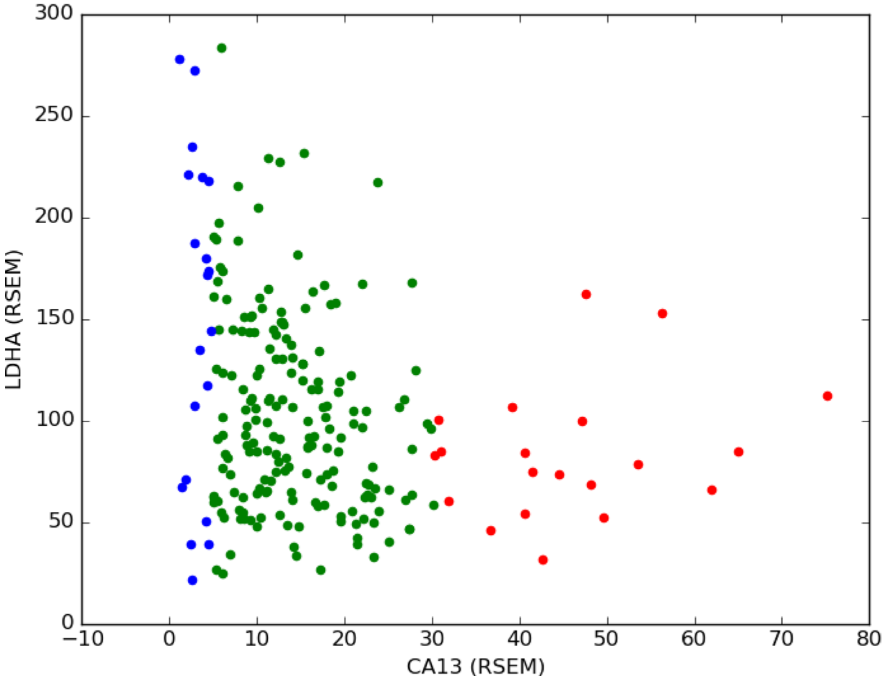

Supplementary Figure 24: Expression of the LDHA gene versus the expression of CA13 in lung tumor samples.

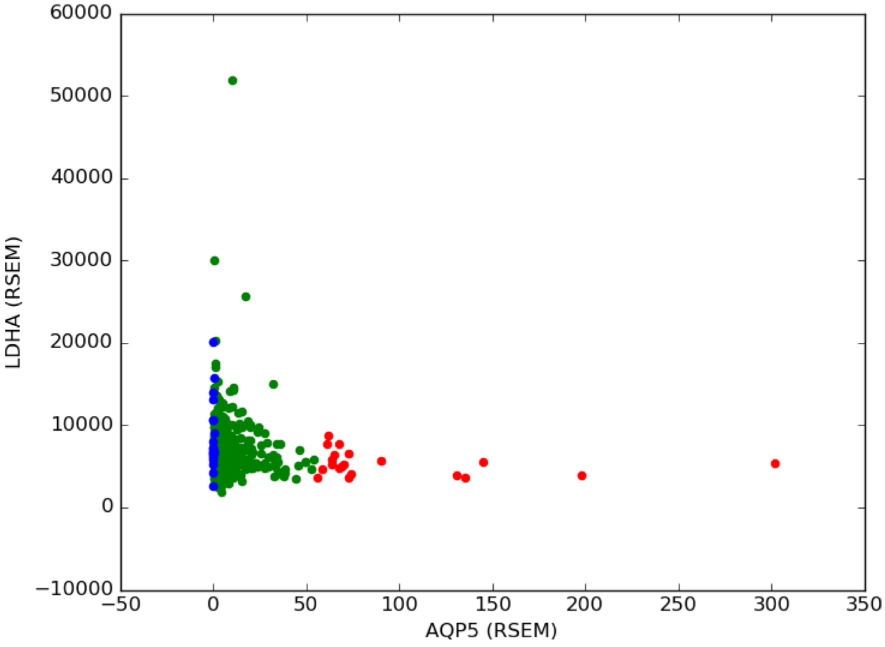

Supplementary Figure 25: Expression of the LDHA gene versus the expression of AQP5 in prostate tumor samples.

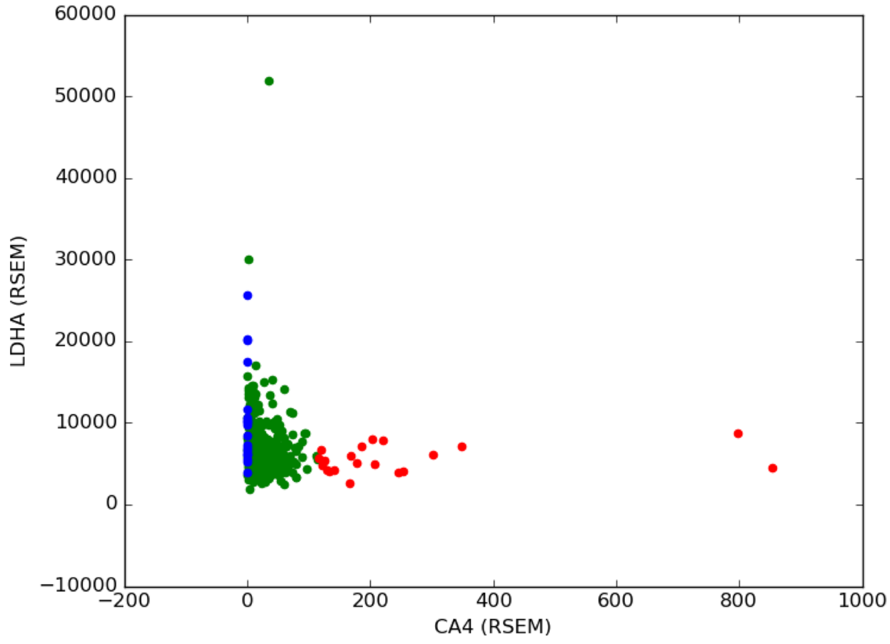

Supplementary Figure 26: Expression of the LDHA gene versus the expression of CA4 in breast tumor samples.

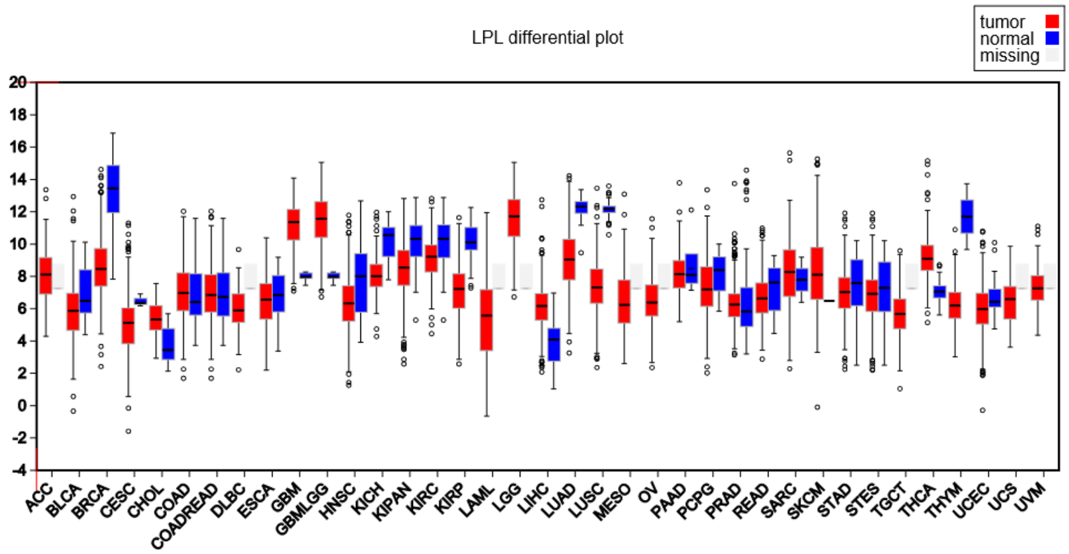

Supplementary Figure 27: Expression of the LPL gene in a panel of different cancer types and normal tissues.

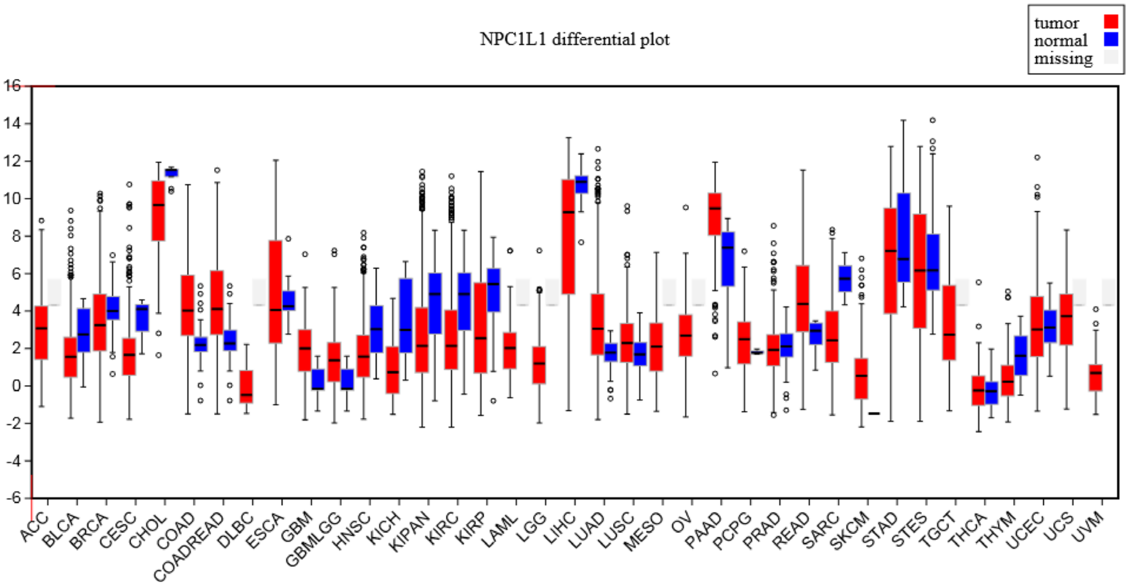

Supplementary Figure 28: Expression of the NPC1L1 gene in a panel of different cancer types and normal tissues.
